# Altering cortical input unmasks synaptic phenotypes in the YAC128 cortico-striatal co-culture model of Huntington disease

**DOI:** 10.1186/s12915-018-0526-3

**Published:** 2018-06-27

**Authors:** Mandi E. Schmidt, Caodu Buren, James P. Mackay, Daphne Cheung, Louisa Dal Cengio, Lynn A. Raymond, Michael R. Hayden

**Affiliations:** 1 Centre for Molecular Medicine and Therapeutics, BC Children’s Hospital Research Institute, University of British Columbia, 950 West 28th Avenue, Vancouver, V5Z 4H4 Canada; 2 Department of Psychiatry and Djavad Mowafaghian Centre for Brain Health, University of British Columbia, 4834-2255 Wesbrook Mall, Vancouver, V6T 1Z3 Canada; 3 Present address: The Hospital for Sick Children, 555 University Avenue, Toronto, M5G 1X8 Canada

**Keywords:** Huntington disease, huntingtin, synapse, spine, dendrite, corticostriatal co-culture, YAC128, DARPP32

## Abstract

**Background:**

Huntington disease (HD) is a fatal neurodegenerative disorder caused by a CAG expansion in the huntingtin (*HTT*) gene, leading to selective and progressive neuronal death predominantly in the striatum. Mutant HTT expression causes dysfunctional cortico-striatal (CS) transmission, loss of CS synapses, and striatal medium spiny neuron (MSN) dendritic spine instability prior to neuronal death. Co-culturing cortical and striatal neurons in vitro promotes the formation of functional CS synapses and is a widely used approach to elucidate pathogenic mechanisms of HD and to validate potential synapto-protective therapies. A number of relevant in vivo synaptic phenotypes from the YAC128 HD mouse model, which expresses full-length transgenic human mutant HTT, are recapitulated in CS co-culture by 21 days in vitro (DIV). However, striatal spine loss, which occurs in HD patients and in vivo animal models, has been observed in YAC128 CS co-culture in some studies but not in others, leading to difficulties in reproducing and interpreting results. Here, we investigated whether differences in the relative proportion of cortical and striatal neurons alter YAC128 synaptic phenotypes in this model.

**Results:**

YAC128 MSNs in 1:1 CS co-culture exhibited impaired dendritic length and complexity compared to wild-type, whereas reducing cortical input using a 1:3 CS ratio revealed a dramatic loss of YAC128 MSN dendritic spines. Chimeric experiments determined that this spine instability was primarily cell autonomous, depending largely on mutant HTT expression in striatal neurons. Moreover, we found that spontaneous electrophysiological MSN activity correlated closely with overall dendritic length, with no differences observed between genotypes in 1:3 co-cultures despite significant YAC128 spine loss. Finally, limiting cortical input with a 1:3 CS ratio impaired the basal survival of YAC128 neurons at DIV21, and this was partially selective for dopamine- and cAMP-regulated phosphoprotein 32-positive MSNs.

**Conclusions:**

Our findings reconcile previous discordant reports of spine loss in this model, and improve the utility and reliability of the CS co-culture for the development of novel therapeutic strategies for HD.

**Electronic supplementary material:**

The online version of this article (10.1186/s12915-018-0526-3) contains supplementary material, which is available to authorized users.

## Background

Huntington disease (HD) is a devastating neurodegenerative disorder caused by a CAG repeat expansion in exon 1 of the huntingtin (*HTT*) gene [1]. The disease is characterized neuropathologically by progressive striatal atrophy and cortical degeneration, leading to impaired cognitive, psychiatric, and motor function [2]. Although overt disease onset occurs during mid-life, human and animal studies have collectively demonstrated that cortico-striatal (CS) synaptic dysfunction occurs early in HD and likely contributes to later neuronal loss [2–5].

Medium spiny neurons (MSNs) make up the vast majority of the striatal neuronal population, and receive a high level of glutamatergic input from the cortex [6, 7]. MSNs are the earliest and most-affected neuronal population in HD, undergoing significant loss of dendritic structure and spines with disease progression in humans and animal models [8–13]. Dysregulated glutamate release at CS synapses in addition to intrinsic MSN properties are hypothesized to ultimately cause selective vulnerability of this cell type [14–17]. However, due to the plasticity of neural connections, CS synaptic dysfunction as well as MSN spine and synapse loss may be therapeutically reversible before neuronal death occurs [4].

The CS neuronal co-culture is a commonly-used in vitro model which consists of cortical and striatal neurons plated homogenously, generally at either a 1:1 or 1:3 cortical:striatal ratio [18]. This method partially recapitulates in vivo circuitry and MSN development, and permits functional CS synapses to be studied in relative isolation from other modulatory neurotransmitters or neuronal inputs [19, 20].

Previous characterization has been performed in 1:1 embryonic CS co-cultures from wild-type (WT) and YAC128 mice (expressing a yeast artificial chromosome containing the full-length human mutant HTT (mHTT) gene encoding 125–128 glutamines [21, 22]) [23, 24]. These studies demonstrated altered extrasynaptic N-methyl D-aspartate (NMDA) receptor function in YAC128 co-cultured MSNs, accompanied by enhanced susceptibility to excitotoxicity as well as reduced CS excitatory synapse activity by 21 days in vitro (DIV), a phenotype undetectable in vivo until 6–7 months of age [15, 25]. Morphology was also evaluated by transfecting MSNs with yellow fluorescent protein (YFP) at the time of plating and, although this analysis showed stunted dendritic complexity in 1:1 co-cultured YAC128 MSNs compared to WT, no difference in spine numbers was observed [23]. This is in stark contrast to studies from another group, in which staining for dopamine- and cAMP-regulated phosphoprotein 32 (DARPP32), a marker of mature MSNs, was used for morphological analysis instead of transfected YFP to show dramatic spine loss in 1:3 CS co-cultured postnatal YAC128 MSNs [13, 26]. The methodological factors underlying the ability to observe this highly relevant HD phenotype remain unknown. DARPP32+ WT MSNs in 1:3 co-culture exhibit less dendritic complexity and fewer spines and synapses than in 1:1 co-culture, indicating that reducing cortical input impairs WT MSN development in vitro [18]*.* However, the impact of altering cortical input in the context of HD has not been evaluated.

In the present study, we have explored whether spine loss is a reproducible feature of HD in this model and investigated the potential methodological factors contributing to the emergence of this phenotype.

## Results

### Reducing cortical input elucidates robust YAC128 MSN spine loss in CS co-culture

**Fig. 1:**
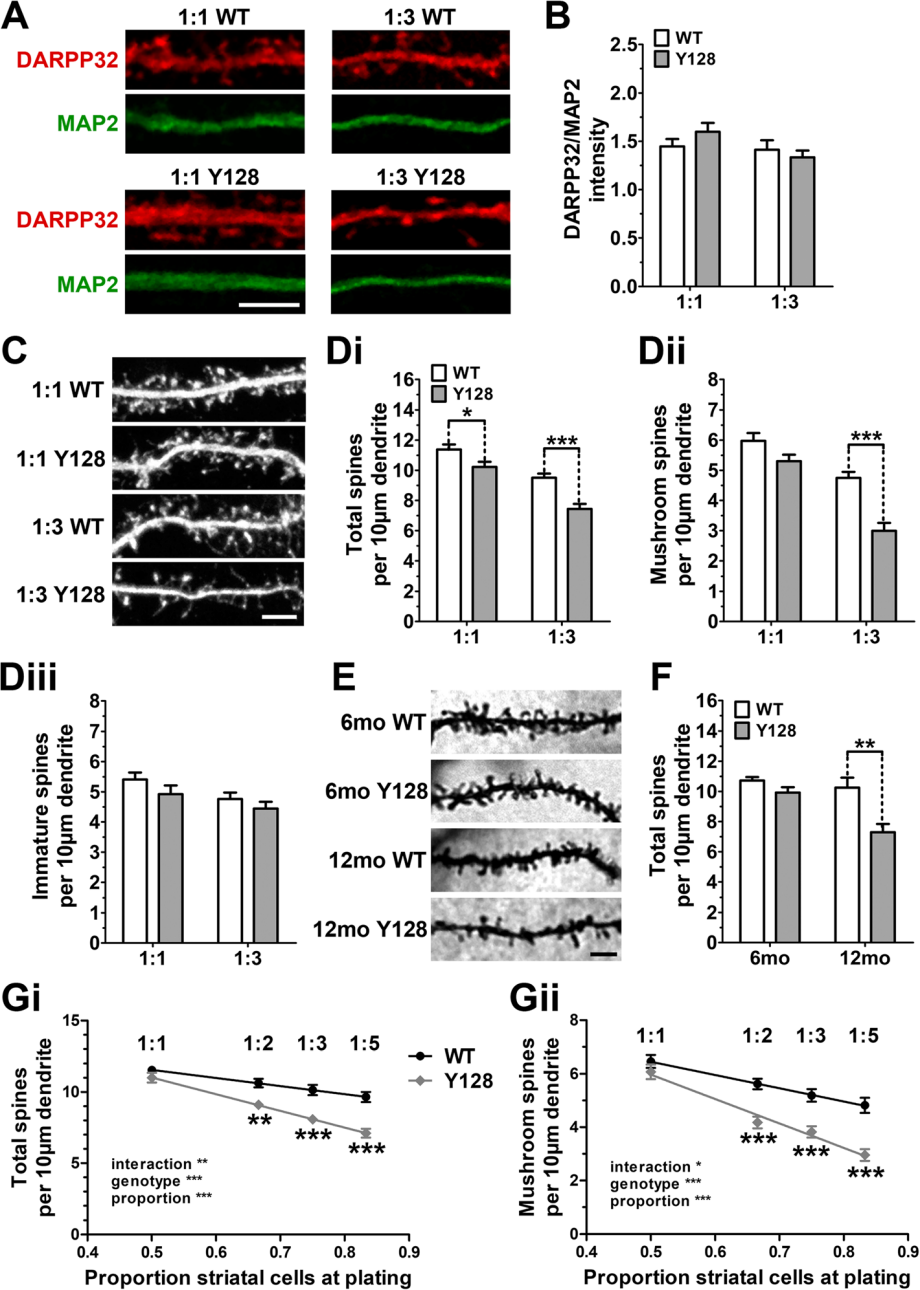
YAC128 MSNs co-cultured with cortical neurons at a 1:3 CS ratio recapitulate in vivo spine loss. WT and YAC128 (Y128) co-cultures were generated at either a 1:1 or 1:3 CS ratio and processed at DIV21 for DARPP32 and MAP2 immunocytochemistry, imaging, and spine analysis in NeuronStudio. (**a**) Sample images of DARPP32- and MAP2-stained dendrites in CS co-culture (scale bar = 5 μm). (**b**) Quantification of DARPP32 staining intensity normalized to MAP2 intensity reveals no differences between genotypes or conditions [*n* = 30(3); two-way ANOVA with Bonferroni post-hoc analysis]. (**c**) Sample images of DARPP32-stained spines on secondary or tertiary dendrites in co-cultured MSNs at higher exposure (scale bar = 5 μm). The differences in numbers of (**Di**) total and (**Dii**) mature mushroom, but not (**Diii**) immature spines, are exacerbated in 1:3 co-cultured YAC128 MSNs [*n* = 32(4); two-way ANOVA with Bonferroni post-hoc analysis; *p < 0.05, ***p < 0.001]. (**e**) Representative Golgi staining of striatal MSNs in vivo (scale bar = 5 μm). (**f**) Golgi analysis confirms that reduced MSN total spine number occurs by 12 months of age in the YAC128 striatum, to a similar degree as in 1:3 co-cultures [*n* = 4–5 6-month-old animals and 3 12-month-old animals per genotype; two-way ANOVA with Bonferroni post-hoc analysis; **p < 0.01]. Individual data values for graph in F are available in Additional file 1. A linear correlation exists between (**Gi**) total and (**Gii**) mushroom spines versus the proportion of striatal cells at plating. A significant interaction occurs between striatal proportion and genotype [*n* = 30(3); two-way ANOVA with Bonferroni post-hoc analysis; *p < 0.05, **p < 0.01, ***p < 0.001]

We first sought to evaluate the effect of altered cortical input on HD-like phenotypes in vitro by culturing WT and YAC128 MSNs with cortical neurons side-by-side at both 1:1 and 1:3 CS ratios, using identical total cell densities. We utilized DARPP32 immunofluorescence staining for MSN morphological analysis in order to remain consistent with the methodology used by Wu et al. [13], as well as to avoid the requirement for YFP nucleofection, which we found to reduce the general health of neuronal cultures. Striatal DARPP32 is decreased in several models of HD, including YAC128 mice [21, 22, 27–31]. To confirm that potentially altered YAC128 DARPP32 expression levels would not interfere with accurate structural analysis, we measured immunofluorescence staining intensity in each culture condition. We co-stained for the dendritic marker microtubule-associated protein 2 (MAP2) and imaged both channels at identical laser intensities across samples. We observed no differences in dendritic DARPP32 intensity normalized to MAP2 intensity (Fig. 1a, b), indicating that MSN DARPP32 expression does not obviously differ between genotypes and that this is an appropriate method for dendritic and spine analysis in this model.

Using this approach, we observed a subtle reduction in MSN total spine density (90% of WT) and a non-significant decrease in mature mushroom spine density (88% of WT) in DIV21 1:1 YAC128 cultures (Fig. 1c, Di, Dii). Remarkably, limiting excitatory input using a 1:3 ratio dramatically enhanced this phenotype, such that the number of total and mature mushroom spines in YAC128 MSNs were reduced to approximately 78% and 63% of WT 1:3 levels, respectively (Fig. 1c, Di, Dii). We did not observe significant differences in the density of immature (stubby, thin, and filopodia) spine types (Fig. 1c, Diii), suggesting a selective impairment in the stability of functionally mature spines.

A previous study using injection of Lucifer yellow fluorescent dye into striatal neurons in brain slices found YAC128 MSN spine loss at 12 months of age, but not at 6 months [13]. We confirmed this finding using a simple Golgi stain method and observe that spine density values and the degree of YAC128 total spine loss at 12 months in vivo (71% of WT) are accurately recapitulated in 1:3 CS co-cultures (Fig. 1e, f and Additional file 1).

To further investigate the relationship between MSN spine density and cortical input, we compared two additional CS ratios (1:2 and 1:5) side-by-side with 1:1 and 1:3 conditions. In this set of experiments, there were no significant genotypic differences in either total or mature mushroom spine densities using a 1:1 ratio. We observed a negative correlation between total and mature mushroom spine densities versus the proportion of striatal cells at the time of plating in both genotypes (Fig. 1Gi, Gii). Interestingly, there was a significant interaction between genotype and CS ratio, with the phenotype becoming more severe with increasing proportion of striatal cells at plating. This indicates that YAC128 MSN spine stability is progressively more sensitive than WT to reduced amounts of cortical input.

Finally, we evaluated the impact of altering the total cell number per well (150,000, 170,000 or 230,000 in 24-well plates), keeping the CS ratio consistent at 1:3. We did not find an effect of initial plating density on the presence or severity of the YAC128 MSN spine phenotype at DIV21 (Additional file 2: Figure S1).

### YAC128 spine instability is predominantly MSN intrinsic

**Fig. 2:**
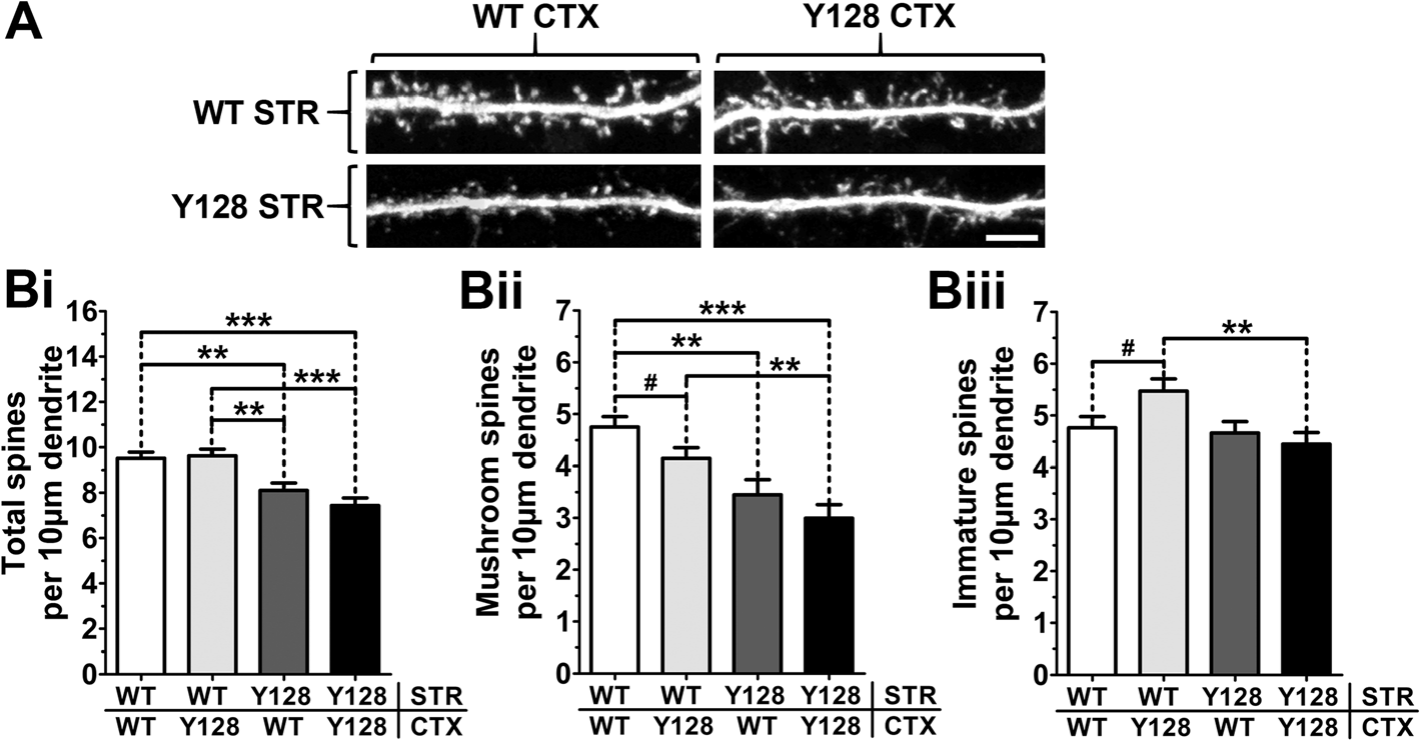
YAC128 spine instability is predominantly MSN intrinsic. WT, YAC128, and chimeric co-cultures generated at a 1:3 CS ratio were processed at DIV21 for DARPP32 immunocytochemistry, imaging, and spine analysis. (**a**) Sample images of DARPP32-stained spines in pure or chimeric co-cultured MSNs (scale bar = 5 μm). (**Bi**) Total spine density values in chimeric cultures are similar to pure cultures of the same MSN genotype. (**Bii**) Mature mushroom and (**Biii**) immature spine numbers are affected by both striatal (STR) and cortical (CTX) mHTT expression [*n* = 32(4); one-way ANOVA with Bonferroni post-hoc analysis; **p < 0.01, ***p < 0.001]. Student’s *t* test was used to compare WT STR/WT CTX and WT STR/Y128 CTX [*n* = 32(4); Student’s *t* test; #*p* < 0.05]

An impaired developmental increase in miniature excitatory post-synaptic current (mEPSC) frequency from DIV14 to DIV21 in 1:1 co-cultured YAC128 MSNs compared to WT was previously reported [23]. Chimeric co-cultures (WT striatal MSNs plated with YAC128 cortical neurons, or vice versa) exhibited an intermediate phenotype, indicating that altered excitatory functional connectivity is partially dependent on mHTT expression in both pre- and post-synaptic compartments [23]. We utilized a similar strategy to determine the relative contribution of each cell type to MSN spine stability in 1:3 co-cultures. We discovered that the difference in total spine numbers between WT and YAC128 was entirely dependent on mHTT expression in the MSN (Fig. 2a, Bi). When we specifically evaluated mature mushroom spines, we found a small contribution from cortical mHTT expression, with chimeric cultures demonstrating a trend to a more intermediate mushroom spine density (Fig. 2a, Bii). When assessed by *t* test, WT MSNs co-cultured with YAC128 cortical neurons had fewer mushroom spines and a greater number of immature spines than those co-cultured with WT cortical neurons, despite similar total spine densities (Fig. 2a, b). Thus, cortical mHTT expression alters the ratio of mature/immature spines in WT neurons. These results suggest that mHTT expression primarily, but not exclusively, in the MSN impairs mechanisms of spine development or stability in response to reduced cortical input.

### Decreasing cortical input masks the YAC128 MSN dendritic complexity phenotype in CS co-culture

**Fig. 3:**
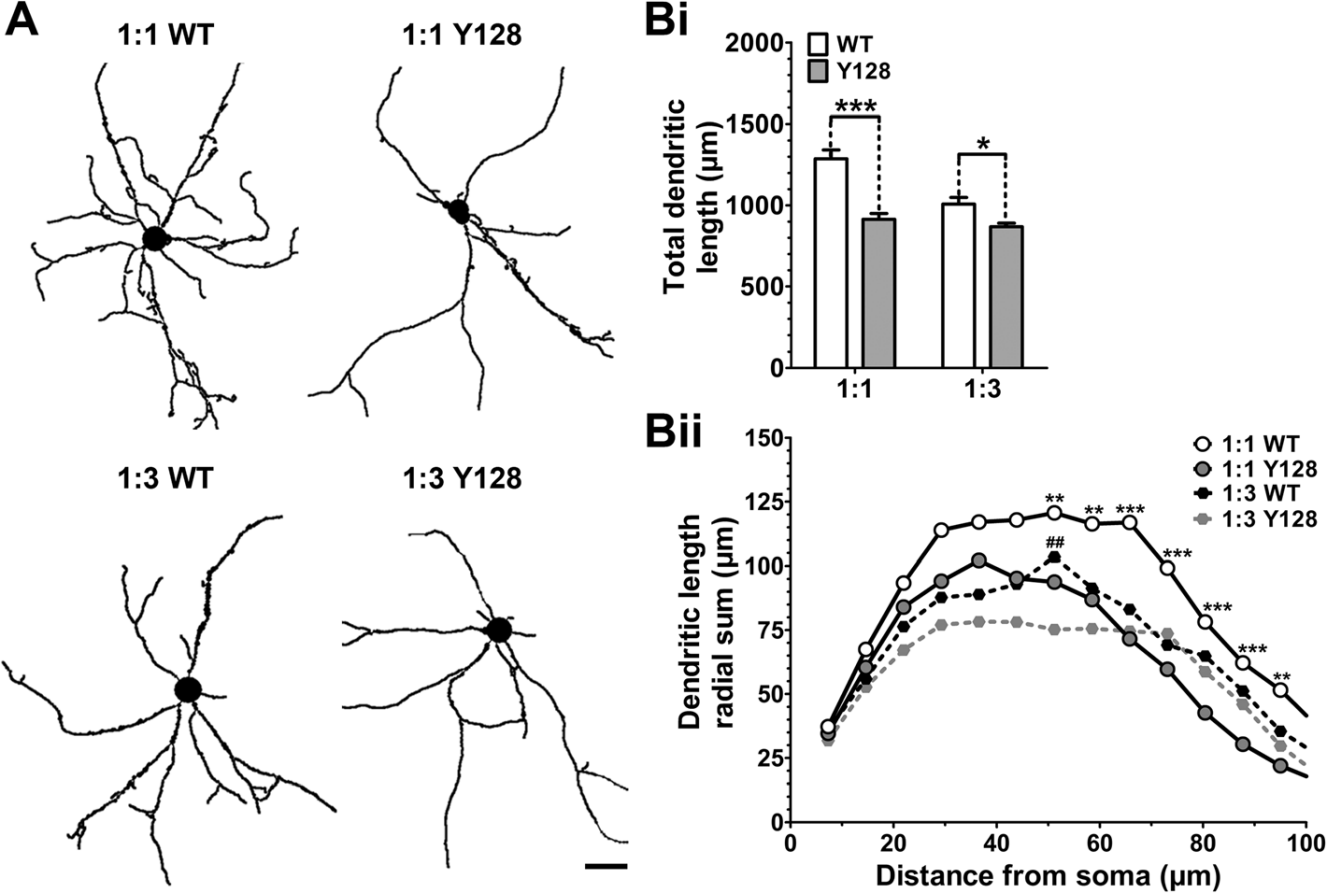
YAC128 MSNs in 1:1 CS co-culture demonstrate reduced dendritic length and complexity. WT and YAC128 co-cultures were generated at either a 1:1 or 1:3 CS ratio and processed at DIV21 for DARPP32 immunocytochemistry, imaging, and dendritic analysis. (**a**) Sample images of MSN dendritic traces generated in NeuronStudio (scale bar = 15 μm). (**Bi**) Total length of the dendritic trace and (**Bii**) complexity by Sholl analysis are significantly reduced in 1:1 YAC128 MSNs compared to WT. Post-hoc statistical significance for Sholl analysis is shown only for WT 1:1 vs. YAC128 1:1 (*) or WT 1:3 vs. YAC128 1:3 (#) comparisons [*n* = 32(4); two-way ANOVA with Bonferroni post-hoc analysis; *p < 0.05, **p < 0.01, ***p < 0.001]

Interestingly, in comparison to MSN spine density, we discovered an opposite effect of CS ratio on MSN dendritic structure by Sholl analysis. A robust impairment in total dendritic length and complexity was observed in DIV21 1:1 co-cultured YAC128 MSNs compared to WT (Fig. 3a, Bi, Bii), in agreement with previous results [23]. However, when a 1:3 CS ratio was utilized, WT MSN dendritic development became impaired, resulting in a much smaller genotypic difference between WT and YAC128 (Fig. 3a, Bi, Bii). Thus, differential elucidation of YAC128 MSN dendritic or spine phenotypes can be achieved by manipulation of the CS ratio.

### YAC128 MSN dendritic and spine phenotypes are developmental in CS co-culture

We next sought to determine at what time-point the identified structural phenotypes are present in CS co-culture. When our DIV21 results were plotted over time along with DIV14 and DIV18 data from the same cultures, we observed that most of the identified YAC128 spine and dendrite alterations were present by DIV18 and all could be attributed to impaired development of YAC128 MSNs after DIV14, at which time there were no discernable phenotypes (Additional file 3: Figure S2 and Additional file 4: Figure S3).

### CS plating ratio influences electrophysiological phenotypes in YAC128 MSNs

**Fig. 4:**
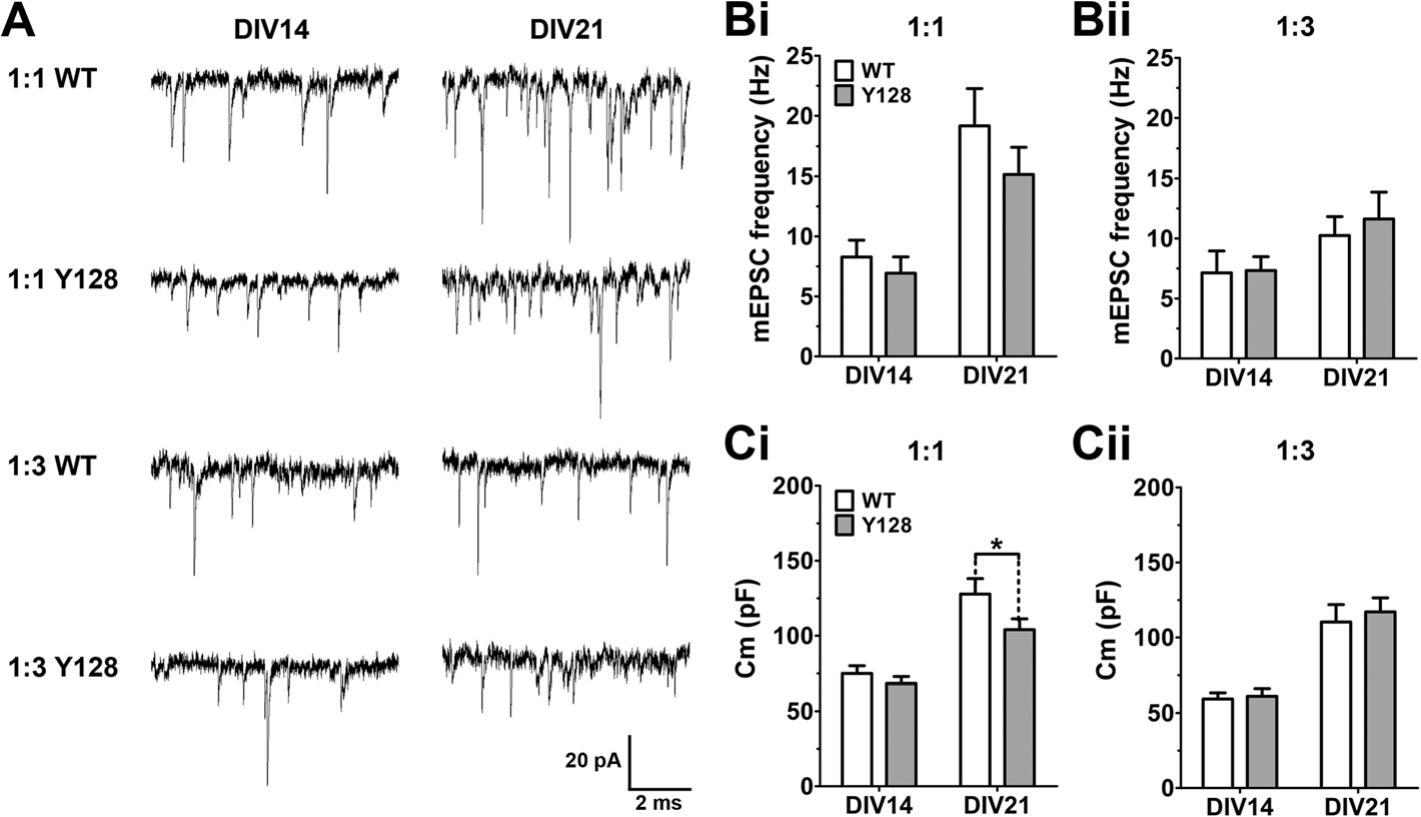
YAC128 MSNs co-cultured at 1:1 exhibit an impaired increase in membrane capacitance with maturation. (**a**) Representative recording traces from WT and YAC128 MSNs in 1:1 or 1:3 co-culture at DIV14 and 21. (**Bi**, **Bii**) mEPSC frequency and (**Ci**, **Cii**) membrane capacitance (Cm) tend to increase with maturation, but a significant genotypic difference was only observed for Cm at DIV21 in 1:1 cultures [*n* = 12–29(3); two-way ANOVA with Bonferroni post-hoc analysis; *p < 0.05]

To determine the functional impact of altering CS ratio, whole-cell patch-clamp electrophysiology was used to record mEPSCs and basal membrane capacitance from MSNs in 1:1 and 1:3 co-cultures at DIV14 and DIV21. Previously published data showed an increase in mEPSC frequency from DIV14 to DIV21 in 1:1 co-cultures, which was blunted in YAC128 MSNs [23]. We observed a similar trend in the current study, although there was no significant genotypic difference between WT and YAC128 at DIV21 (Fig. 4a, Bi). However, when a 1:3 ratio was utilized, there was only a small increase in mEPSC frequency from DIV14 to DIV21 for both WT and YAC128 such that there was no longer a trend to a difference between genotypes at DIV21 (Fig. 4a, Bii). This is consistent with a prior study which found reduced mEPSC frequency in DIV18 1:3 co-cultured WT MSNs compared to 1:1 [18]. Membrane capacitance, a measure of overall MSN size, increased with time in all culture conditions (Fig. 4Ci, Cii). However, the increase in 1:1 WT MSNs was more dramatic than in 1:1 YAC128 MSNs, elucidating a significant genotypic difference at DIV21, which was not observed in 1:3 co-cultures (Fig. 4Ci, Cii). This correlates well with our observation of a greater difference in dendritic arbor size and complexity between genotypes using a 1:1 CS ratio. These findings indicate that the previously published YAC128 mEPSC frequency and capacitance phenotypes are also CS ratio-dependent and that overall MSN functional connectivity correlates more closely with dendritic development than with spine density.

### Reducing cortical input promotes neuronal death in YAC128 CS co-culture

**Fig. 5:**
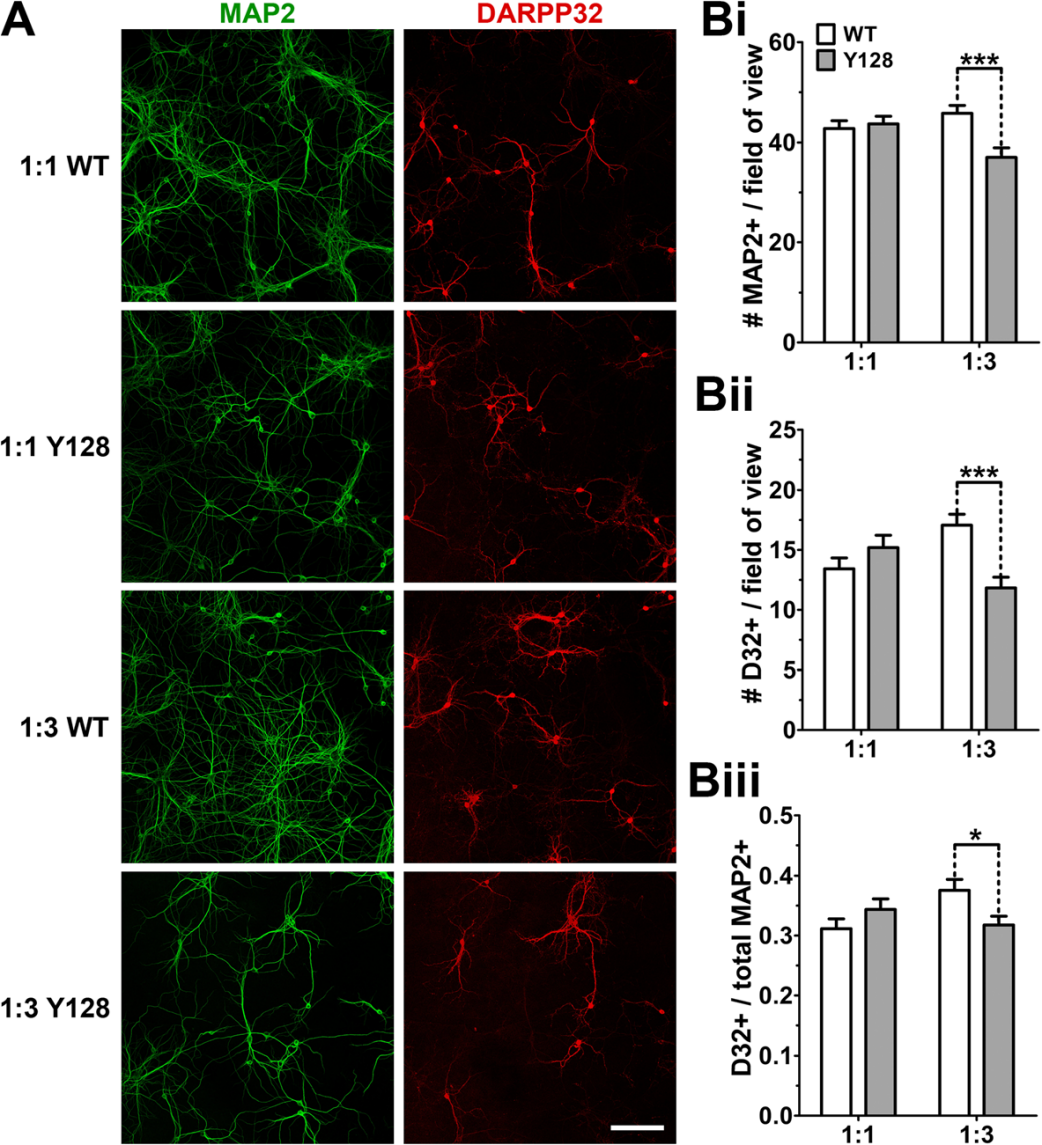
Neuronal survival is compromised in YAC128 1:3 CS co-cultures. DIV21 WT and YAC128 co-cultures were fixed at DIV21 and stained for MAP2 and DARPP32 (D32). (**a**) Sample fields of view at 20X objective (scale bar = 100 μm). The numbers of (**Bi**) MAP2+ and (**Bii**) DARPP32+ neurons per field of view are reduced in YAC128 1:3 co-cultures. (**Biii**) The proportion of DARPP32+ neurons (# DARPP32+ divided by # MAP2+) surviving at DIV21 is also significantly lower in YAC128 1:3 co-cultures [*n* = 30 fields of view from three independent cultures; two-way ANOVA with Bonferroni post-hoc analysis; *p < 0.05, ***p < 0.001]

Previously, WT neurons (both cortical and striatal DARPP32+ MSNs) exhibited reduced basal survival at DIV18 when co-cultured at a 1:3 CS ratio versus 1:1 [18]. We used a similar approach to compare neuronal survival in DIV21 WT and YAC128 neurons at both CS ratios. We found significantly reduced survival of all neurons (MAP2+) as well as DARPP32+ MSNs in YAC128 1:3 co-cultures compared to WT 1:3 (Fig. 5a, Bi, Bii), despite being initially plated at identical live cell density. When we calculated the proportion of surviving MAP2+ neurons that were also DARPP32+, we found that neuronal loss in YAC128 1:3 co-cultures was partially selective for this cell population (Fig. 5a, Biii). This reveals an additional CS ratio-dependent co-culture phenotype that may be useful for future studies of mutant HTT-induced neuronal death.

### In vitro DiOlistic labeling reveals increased thin spines and reduced mushroom spine head size in mono-cultured YAC128 cortical neurons

Although striatal MSNs are the most severely-affected cell type in HD, there is evidence that mHTT causes neuronal and synaptic dysfunction in other brain regions as well, including the cortex and thalamus [5, 32, 33]. Thus, it may be desirable to utilize modified culture models for the study of these neuronal populations. For example, a YAC128 thalamo-striatal co-culture model was recently used to demonstrate mHTT-induced thalamo-striatal synaptic dysfunction [32].

We attempted to combine a previously reported in vitro 1,1′-dioctadecyl-3,3,3′,3’-tetramethylindocarbocyanine perchlorate (DiI) DiOlistic dye labeling protocol [34] with immunocytochemistry for glutamatergic markers in order to perform spine analysis on cortical neurons in CS co-culture. However, permeabilization of DiI-stained cells for internal staining resulted in the release of DiI from cell membranes and poor filling of spines. Instead, we generated WT and YAC128 pure cortical monocultures for DiI spine analysis at DIV21. We did not observe any differences in total, mushroom, or stubby spine densities between genotypes, although there was an increased number of thin spines in YAC128 cortical neurons (Additional file 5: Figure S4). Interestingly, we observed a significant 7% reduction in the diameter of YAC128 mushroom spines (Additional file 5: Figure S4), indicating that subtle dysfunction in cortical neurons may also exist in vitro, which could contribute to CS synaptic alterations.

## Discussion

### Optimization of the CS co-culture for elucidation of YAC128 synaptic phenotypes

The CS co-culture has become an attractive methodological option for the isolated study of both physiological and pathogenic mechanisms of CS synaptic function. This model allows direct assessment of neuronal morphology and synaptic transmission and can be used to quickly answer specific questions that are difficult to investigate using in vivo animal models. Mutant HTT-expressing YAC128 CS co-cultures recapitulate many relevant in vivo synaptic phenotypes by 21 days in vitro [23], highlighting the practicality of this model as a primary tool for therapeutic target validation*.*

Spine instability, hypothesized to contribute to neuronal dysfunction in HD and other neurodegenerative disorders, has been observed in YAC128 MSNs in CS co-culture in some studies, but not in others [13, 23, 26]. Recently, altering CS plating ratio was found to affect a number of functional and morphological characteristics of WT MSNs [18], leading us to hypothesize that modifying cortical input in YAC128 CS co-cultures may elucidate or exacerbate synaptic phenotypes, thus enhancing the utility of this culture system for HD research. In the present study, we have clearly shown that modifying CS ratio in co-culture differentially elucidates YAC128 MSN synaptic phenotypes (summarized in Table 1). For future studies of therapeutic strategies to modify neurite growth or stability in HD, a 1:1 CS ratio is recommended. Conversely, for evaluation of potential neuroprotective or spine-stabilizing therapies, a 1:3 CS ratio is ideal, as this accurately recapitulates YAC128 age-associated in vivo MSN spine loss and neuronal death.

**Table 1:** Optimal CS ratios to elucidate YAC128 MSN phenotypes in co-culture

YAC128 MSN Phenotype	Optimal CS ratio
Spine loss	1:3
Impaired neuronal survival	1:3
Decreased dendritic length/complexity	1:1
Reduced mEPSC frequency	1:1
Reduced membrane capacitance	1:1

### Intrinsic versus extrinsic effects of mHTT on MSN spine stability

Our result showing that decreasing the proportion of cortical neurons in CS co-culture promotes spine instability in YAC128 MSNs raises the interesting possibility that spine loss with disease progression in vivo is partially due to reduced cortical input. Indeed, studies support the hypothesis that progressive CS disconnect in HD results in loss of cortical excitatory and trophic support to MSNs over time and striatal degeneration [3, 35]. However, in seeming contradiction, our experiments using chimeric cultures demonstrate that YAC128 MSN spine instability is primarily cell autonomous (Fig. 2). We propose that mHTT expression in MSNs renders spines intrinsically more sensitive to low levels of cortical support, causing this phenotype to only emerge in the presence of reduced cortical input. There is evidence that depletion of endoplasmic reticulum calcium stores and consequent enhanced store-operated calcium entry in YAC128 MSNs contributes to spine loss in CS co-culture [13]. It is possible that reducing glutamatergic input with a 1:3 CS ratio exacerbates endoplasmic reticulum store depletion in YAC128 MSNs by limiting normal activity-induced extracellular calcium influx, which might subsequently promote more dramatic spine loss.

A recent study investigated the contribution of cortical or striatal mHTT to synaptic dysfunction by crossing region-specific Cre-expressing mice to the BACHD mouse model (expressing a bacterial artificial chromosome containing the full-length human mutant huntingtin gene with 97 mixed CAA-CAG repeats [PMID: 18550760]) [36]. It was discovered that mHTT expression predominantly in the cortex was required for altered synaptic protein levels and reduced spontaneous EPSC frequency in the striatum of aged BACHD mice, while impaired evoked NMDA current was dependent on mHTT expression in both the striatum and cortex [36]. A follow-up study found improvement in striatal activity patterns and behavioral phenotypes in response to mHTT reduction in the cortex of BACHD mice [37]. Although our results in the present study showed that total spine density was determined entirely by mHTT expression in striatal neurons, we did observe a small effect of cortical expression on mushroom spine numbers. In particular, WT MSNs co-cultured with WT cortical neurons possessed similar total spine density as those co-cultured with YAC128 cortical neurons, but we observed fewer mushroom spines and a greater number of immature spines in MSNs from the chimeric cultures (Fig. 2). Since mature and immature spines would be expected to have different functional properties, this indicates that cortical mHTT expression may contribute to altered CS synaptic readouts. In further support of this hypothesis, we also report subtle spine morphology alterations in monocultured YAC128 cortical neurons (Additional file 5: Figure S4).

### Spine and dendritic alterations in HD patients and animal models

Early reports using Golgi staining of postmortem HD patient brain samples demonstrated both proliferative and degenerative morphological alterations in striatal MSNs [8, 38]. These included an increase in the number and size of dendritic spines as well as altered dendritic branching in early stage (grade 2) HD [8]. In advanced HD brains, smaller dendritic arbors, spine loss, and dendritic swellings were observed [8]. It is hypothesized that early proliferative changes could reflect activation of compensatory mechanisms in response to synaptic dysfunction, which eventually become overwhelmed with disease progression and age. This is supported by observations of increased glutamate transmission onto striatal neurons at early time points in the YAC128 and BACHD mouse models, followed by reduced transmission at later ages [15, 39].

Multiple mouse models of HD recapitulate the structural degeneration observed in advanced HD brains. Both MSNs and cortical pyramidal neurons in R6/1 mice (N-terminal HTT fragment mouse model of HD with 116 CAG repeats [40]) exhibit reduced spine density and spine length at symptomatic ages, and a later study also reported thinner apical dendrites in the somatosensory cortex [12, 41]. Similarly, symptomatic R6/2 mice (N-terminal HTT fragment mouse model of HD with 144–150 CAG repeats [40]) demonstrate MSN spine loss in addition to thinner dendritic shafts [9, 42]. Studies in full-length mHTT models, including mHTT knock-in and BACHD mice, have also shown loss of dendritic spines in HD MSNs [43, 44]. Although we and others observed YAC128 MSN total spine loss at 12 months of age, but not at 6 months (Fig. 1f) [13], a 15% reduction in secondary and tertiary dendrite spine density at 3 months of age has been reported [11], as well as diminished excitatory CS activity at 6–7 months [15, 25]. Thus, an effect of mHTT expression on spines and synapses is present in YAC128 mice but may be too subtle at early ages to be detected reliably by structural analysis in vivo.

### Developmental synaptic phenotypes in YAC128 CS co-culture

We found that all of the identified DIV21 phenotypes were due to impaired development of YAC128 MSNs after DIV14 (Additional file 3: Figure S2 and Additional file 4: Figure S3). In vivo, MSN spines and dendrites develop normally in WT and YAC128 animals when assessed by Golgi staining at 1 month of age [17]. Thus, our observation of developmental phenotypes in CS co-culture suggests that impaired synaptic function occurs early in vitro, before MSNs have reached a mature state. This is in agreement with previous work showing an impaired developmental increase in mEPSC frequency and stunted dendritic development after DIV14 using YFP transfection in co-cultured YAC128 MSNs [23]. However, our results are discordant with a recent study showing degenerative spine loss from DIV14 to DIV21 in YAC128 CS co-cultured MSNs [13]. Differences in culture methodology might explain why Wu et al. [13] observed a degenerative phenotype and we did not. If our culturing conditions were inherently more stressful to the neurons, their maturation by DIV14 may have been impaired, such that synaptic dysfunction occurred before spines or dendrites were fully developed. Alternatively, the use of postnatal cultures in Wu et al. [13] might have promoted earlier maturation of MSNs by DIV14, either due to the later developmental age used or the presence of a greater number of supporting glial cells in the postnatal brain [45]. The existence of YAC128 dendritic and spine phenotypes at DIV18 but not at DIV14 is advantageous as it allows for in vitro testing of both preventative therapies (i.e., from DIV14–21) or strategies aimed at phenotype reversal (i.e., from DIV18–21).

### Functional impact of altering cortical input in CS co-culture

Our electrophysiological results demonstrate that a 1:1 CS ratio is critical for the emergence of a YAC128 mEPSC frequency or membrane capacitance phenotype, which tend to correlate with total dendritic length (summarized in Table 1). Surprisingly, 1:3 co-cultured YAC128 MSNs had similar mEPSC frequencies to 1:3 WT MSNs, despite exhibiting significantly impaired spine stability. This finding raises the possibility that YAC128 cortical or striatal neurons in 1:3 cultures undergo compensatory upregulation of spontaneous CS activity, potentially by increasing cortical glutamate release. It is also plausible that some of the additional spines on WT 1:3 MSNs possess NMDA receptor-containing silent synapses, which would not be active in our electrophysiological recording conditions, and thus may not result in an increased mEPSC frequency compared to YAC128 [46]. Alternatively, YAC128 1:3 MSNs could conceivably contain a higher number of active shaft synapses, which likely constitute a large proportion of synapses in cultured neurons [47], and may be detected by electrophysiological recording, but would not be identifiable through spine analysis. One caveat in our interpretation of these results is that identification of MSNs for electrophysiological recording in CS co-culture requires a striatal YFP transfection step at the time of plating [23, 24], which could reduce overall culture health and thus impact the level of spontaneous activity observed. Furthermore, it is possible that YFP transfection and DARPP32 staining disproportionately identify MSN populations of different subtypes or maturity, leading to inconsistencies when comparing data obtained with each method.

### Selective, age-associated loss of DARPP32+ MSNs in the YAC128 mouse model

Previous analysis of DARPP32+ MSN survival in WT CS co-cultures demonstrated that, despite a 50% higher striatal plating density in 1:3 versus 1:1 cultures, the number of DARPP32+ cells at DIV18 was similar, suggesting selective vulnerability of this cell type [18]. In the present study, the density and proportion of WT DARPP32+ MSNs in 1:3 conditions at DIV21 increased by 27% and 21%, respectively, compared to 1:1, although this was still less than the expected 50% increase (Fig. 5). It is possible that DARPP32 expression was higher after longer maturation to DIV21 in our study, potentially improving the sensitivity of this readout compared to the DIV18 study. Interestingly, YAC128 DARPP32+ MSNs in 1:3 CS co-culture exhibit reduced survival compared to WT when assessed at DIV21 (Fig. 5). This correlates well with our previously established findings of striatal volume loss and reduced DARPP32+ MSN cell counts in 12-month old YAC128 brains [22, 27–29], as well as decreased DARPP32 protein and mRNA levels at 10 months of age [21]. These in vivo alterations are associated with behavioral impairments that are less severe or not observable at earlier ages [22, 48]. Thus, we have enhanced our in vitro CS co-culture model to recapitulate age-associated MSN loss without the use of any acute stressors, such as glutamate, to induce cell death. This will prospectively be useful for preclinical testing of neuroprotective therapeutic approaches in a more representative model of chronic disease.

## Conclusions

We have optimized the CS co-culture system for broader and more reliable use in HD research and show that intrinsic MSN spine stability is highly sensitive to cortical input, thus providing both a clear explanation for inconsistent results from previous studies and a strategy to generate reproducible and disease-relevant findings in the future. The ability to observe a consistent spine phenotype in vitro is likely to be useful for preclinical HD drug development, because spine loss in YAC128 MSNs is dynamic, such that it can be modulated over relatively short periods of time [13, 26]. This provides a sensitive experimental readout for future studies of mHTT-induced synaptic dysfunction. Furthermore, the techniques we have utilized for morphological analysis are accessible, easy to establish and can be used to generate results quickly compared to in vivo studies. Ultimately, our findings demonstrate that the CS co-culture system is amenable to modifications that allow differential elucidation of HD-like phenotypes in vitro, and provide a useful tool for future studies on mechanisms of synaptic dysfunction in HD.

## Methods

### Neuronal culture

Timed pregnancies were set up by mating wild-type FVB/N female mice with YAC128 (line 53) males. At E17.5, embryos were removed from anesthetized mothers and brains were extracted and stored in a Hibernate solution (Hibernate-E supplemented with L-glutamine and B27; Gibco) overnight while excess embryonic tissue was genotyped. Cortical and striatal tissues from both male and female embryos were dissected separately the following day in ice-cold Hank’s Balanced Salt Solution, gently dissociated with a P1000 pipette, and incubated in 0.05% trypsin-EDTA (Gibco) at 37 °C for 8 min. Cells were further dissociated with a short DNase treatment followed by resuspension in complete neurobasal medium (NBM; supplemented with B27, penicillin-streptomycin, and L-glutamine; Gibco). Neurons from appropriate genotypes were combined at a 1:1, 1:2, 1:3, or 1:5 cortico:striatal ratio and plated on 12 mm glass coverslips (Marienfeld Superior) in 24-well plates at a final density of 170,000 cells per well in 1 mL of complete NBM. Prior to plating, coverslips were treated overnight with 6 N hydrochloric acid, washed thoroughly with sterile water and 70% ethanol, transferred to culture plates, and coated with sterile-filtered 50 μg/mL of poly-D-lysine hydrobromide (Sigma; P7886) in water overnight at room temperature. Coverslips were washed four times with sterile water and allowed to air dry before plating. For electrophysiological experiments, YFP was transfected into striatal neurons at the time of plating to allow for MSN identification. Approximately 2 million striatal neurons were suspended in 100 μL of electroporation solution (Mirus Bio) prior to the final plating step, mixed with 2 μg of DNA (YFP on a β-actin promoter; a gift from A.M. Craig, University of British Columbia), and nucleofected (Amaxa Nucleofector, Lonza Bio, program 05). Cells were diluted and plated in 500 μL of 10% fetal bovine serum/DMEM. Media was replaced with 500 μL of complete NBM after 4 h, and topped up to 1 mL the following day. All cultures were supplemented with fresh NBM complete (20% well volume) every 3–7 days until fixation of coverslips at DIV14, 18, or 21.

### Immunocytochemistry

Neurons on coverslips were fixed in 4% paraformaldehyde (PFA)/phosphate buffered saline (PBS) for 15 min at room temperature (RT), incubated in ice-cold methanol for 5 min at −20 °C, permeabilized in 0.03% Triton-X/PBS for 5 min at RT, and blocked for 30 min at RT in 0.2% gelatin/PBS. Coverslips were incubated with primary antibody against DARPP32 (rat anti-DARPP32; R&D Systems Cat# MAB4230; RRID:AB_2169021; 1:500) and MAP2 (mouse anti-MAP2; Invitrogen Cat# MA5–12823; RRID:AB_10982160; 1:200) in blocking buffer overnight at 4 °C, washed in PBS, stained with secondary antibodies against rat IgG (Alexa Fluor 568 goat anti-rat IgG; Invitrogen Cat# A-11077; RRID:AB_141874; 1:500) or against mouse IgG (Alexa Fluor 488 goat anti-mouse IgG; Invitrogen Cat# A-11001; RRID:AB_2534069; 1:500) for 1.5 h at RT, washed in PBS, and mounted on slides using Prolong Gold Antifade Reagent with DAPI (Invitrogen). For spine and dendrite analysis, fluorescence images were acquired using a Leica TCS SP8 confocal laser scanning microscope at 63X objective magnification. Samples from different groups were interleaved and the researcher was blinded to experimental conditions during imaging and analysis. Image stacks of Z-step size of 60 μm were converted to 2D in Image J using the maximum intensity Z-projection function. Images were then background subtracted with a rolling ball radius of 35 pixels and de-speckled. Images were imported into NeuronStudio (Version 0.9.92) for semi-automated Sholl analysis as well as spine characterization using a minimum of three representative secondary or tertiary dendritic segments per cell. For analysis of DARPP32 and MAP2 staining intensity and cell survival counts, random fields of view were imaged at 20X objective magnification using identical laser intensities across samples. The number of MAP2+ or DARPP32+ with healthy nuclei in each field of view were counted, and staining intensity was measured within multiple secondary or tertiary dendrite regions from each neuron selected for analysis.

### DiOlistic labeling of cortical neurons

Cortical neurons were labeled in vitro with DiI stain (Invitrogen Cat# D282) as previously described [34], with minor alterations. Briefly, DIV21 cortical cultures were fixed in 2% PFA/PBS for 15 min at RT. Then, 15–20 DiI crystals were sprinkled on top of coverslips, and a small volume of PBS was added to prevent cells from drying out. Coverslips were incubated in the dark for 10 min at RT, followed by thorough PBS washing to remove crystals and incubation in the dark for an additional 6 h in PBS. Coverslips were rinsed again in PBS and mounted on slides. Imaging and spine analysis were performed as described above with an excitation wavelength of 549 nm.

### Golgi-Cox staining

Six- or 12-month-old mice were perfused with 2% PFA/2% glutaraldehyde/PBS, post-fixed in the same solution overnight at 4 °C and processed as previously described [49], with minor alterations. Briefly, brains were washed in PBS, incubated in Golgi-Cox solution (1% potassium dichromate, 1% mercuric chloride, 0.8% potassium chromate) for 5 days, and transferred to 30% sucrose/PBS. Then, 100 μm sections were cut on a vibratome and mounted on slides, which were dried overnight, washed in ddH_2_O, incubated in 20% ammonium hydroxide for 10 min, washed in ddH_2_O, passed through ascending grades of alcohol, and placed in xylene for 5 min. Coverslips were mounted on top of sections with Cytoseal mounting medium (Thermo Scientific). Transmitted light images were acquired with a Leica TCS SP8 confocal laser scanning microscope and a 63X objective lens. Images were imported into NeuronStudio and spines on dendritic segments from at least 15–20 neurons per animal were semi-automatically analyzed.

### Electrophysiology

Whole-cell patch-clamp electrophysiology was conducted as previously described [23]. Briefly, an Axon Instrument Axopatch B200B amplifier and pClamp 10.2 software (Molecular Devices) were used to collect data under the voltage-clamp mode. Culture coverslips were perfused in a recording chamber with external recording solution containing picrotoxin and tetrodotoxin [23]. mEPSCs were recorded in YFP-positive neurons at a holding membrane potential of –70 mV with the recording pipettes filled with K-gluconate internal solution [23]. Membrane capacitances were measured within 2 min of patching each cell, and at least 30 synaptic events were analyzed per cell with Clampfit 10.2 or 10.7.

### Data analysis

All data is presented as mean ± SEM. Statistical analysis and graph generation were performed using GraphPad Prism 5, and figures were created in Adobe Photoshop CS5. *n* values for all experiments are recorded as the total number of cells analyzed, with the number of independent cultures in parentheses. Student’s *t* test or one- or two-way ANOVA statistical tests with Bonferroni post-hoc analysis were used for all experiments.

## Additional files

Additional file 1: Individual data values. Individual values for data with *n* < 6. (XLSX 12 kb)

Additional file 2: **Figure S1.** Initial plating density does not impact the presence or severity of YAC128 MSN spine instability. WT and YAC128 co-cultures were generated at a 1:3 CS ratio and plated at three different total cell numbers per well (150,000, 170,000, or 230,000 in 24-well plates). Coverslips were fixed at DIV21 and processed for DARPP32 immunocytochemistry, imaging, and spine analysis. (A) Sample images of DARPP32-stained spines on secondary or tertiary MSN dendrites (scale bar = 5 μm). There was no effect of initial plating density on YAC128 (Bi) total or (Bii) mature mushroom spine density phenotypes [*n* = 20(2); two-way ANOVA with Bonferroni post-hoc analysis; *p < 0.05, **p < 0.01, ***p < 0.001]. (TIF 423 kb)

Additional file 3: **Figure S2.** Reduced spine density in co-cultured YAC128 MSNs is a developmental phenotype. WT and YAC128 co-cultures were generated at either a 1:1 or 1:3 CS ratio and processed at DIV14, 18, or 21 for DARPP32 immunocytochemistry, imaging, and spine analysis. (A) Sample images of DARPP32-stained spines on secondary or tertiary MSN dendrites (scale bar = 5 μm). A developmental increase in (Bi) total and (Bii) mature mushroom spine numbers is impaired after DIV14 in co-cultured YAC128 MSNs compared to WT [*n* = 32(4); two-way ANOVA with Bonferroni post-hoc analysis; *p < 0.05, **p < 0.01, ***p < 0.001]. (TIF 988 kb)

Additional file 4: **Figure S3.** Reduced dendritic length and complexity in co-cultured YAC128 MSNs are developmental phenotypes. WT and YAC128 co-cultures were generated at either a 1:1 or 1:3 CS ratio and processed at DIV14, 18, and 21 for DARPP32 immunocytochemistry, imaging, and dendritic analysis. (A) Sample images of MSN dendritic traces generated in NeuronStudio (scale bar = 15 μm). A developmental increase in (Bi, Bii, Biii) dendritic complexity by Sholl analysis and (Biv) total dendritic length are impaired after DIV14 in co-cultured YAC128 MSNs compared to WT. Post-hoc statistical significance for Sholl analysis is shown only for WT 1:1 vs. YAC128 1:1 (*) or WT 1:3 vs. YAC128 1:3 (#) comparisons [*n* = 32(4); two-way ANOVA with Bonferroni post-hoc analysis; *p < 0.05, **p < 0.01, ***p < 0.001]. (TIF 875 kb)

Additional file 5: **Figure S4.** Increased thin spine density and reduced mushroom spine head diameter in DIV21 YAC128 cortical neurons. WT and YAC128 pure cortical cultures were fixed at DIV21 and subjected to in vitro DiI DiOlistic dye labeling for spine analysis. (A) Sample images of DiI-stained spines on cortical dendrites (scale bar = 5 μm). No significant differences in (Bi) total, (Bii) mushroom, or (Biii) stubby spine densities were observed in YAC128 cortical neurons. (Biv) Increased thin spine density and (Bv) reduced mushroom spine head diameter were measured in YAC128 cortical neurons compared to WT [*n* = 30(3); Student’s *t* test; *p < 0.05]. (TIF 288 kb)

## Abbreviations

CS: cortico-striatal
DARPP32: dopamine- and cyclic AMP-regulated phosphoprotein 32
DiI: 1,1′-dioctadecyl-3,3,3′,3’-tetramethylindocarbocyanine perchlorate
DIV: days in vitro
HD: Huntington disease
HTT: huntingtin
MAP2: microtubule-associated protein 2
mEPSC: miniature excitatory postsynaptic current
mHTT: mutant huntingtin
MSN: medium spiny neuron
NBM: neurobasal medium
NMDA: N-methyl D-aspartate
PBS: phosphate buffered saline
PFA: paraformaldehyde
RT: room temperature
WT: wild-type
YFP: yellow fluorescent protein

## References

[1] Huntington’s Disease Collaborative Research Group (1993). A novel gene containing a trinucleotide repeat that is expanded and unstable on Huntington’s disease chromosomes. The Huntington’s Disease Collaborative Research Group. Cell.

[2] Raymond LA, André VM, Cepeda C, Gladding CM, Milnerwood AJ, Levine MS (2011). Pathophysiology of Huntington’s disease: time-dependent alterations in synaptic and receptor function. Neuroscience.

[3] Cepeda C, Wu N, André VM, Cummings DM, Levine MS (2007). The corticostriatal pathway in Huntington’s disease. Prog Neurobiol.

[4] Tyebji S, Hannan AJ (2017). Synaptopathic mechanisms of neurodegeneration and dementia: Insights from Huntington’s disease. Prog Neurobiol.

[5] Estrada-Sánchez AM, Rebec GV (2013). Role of cerebral cortex in the neuropathology of Huntington’s disease. Front Neural Circuits.

[6] Kemp JM, Powell TP (1971). The structure of the caudate nucleus of the cat: light and electron microscopy. Philos Trans R Soc Lond Ser B Biol Sci.

[7] McGeorge AJ, Faull RLM (1989). The organization of the projection from the cerebral cortex to the striatum in the rat. Neuroscience.

[8] Ferrante RJ, Kowall NW, Richardson EP (1991). Proliferative and degenerative changes in striatal spiny neurons in Huntington’s disease: a combined study using the section-Golgi method and calbindin D28k immunocytochemistry. J Neurosci.

[9] Klapstein GJ, Fisher RS, Zanjani H, Cepeda C, Jokel ES, Chesselet MF (2001). Electrophysiological and morphological changes in striatal spiny neurons in R6/2 Huntington’s disease transgenic mice. J Neurophysiol.

[10] Laforet GA, Sapp E, Chase K, McIntyre C, Boyce FM, Campbell M (2001). Changes in cortical and striatal neurons predict behavioral and electrophysiological abnormalities in a transgenic murine model of Huntington’s disease. J Neurosci.

[11] Marco S, Giralt A, Petrovic MM, Pouladi MA, Martínez-Turrillas R, Martínez-Hernández J (2013). Suppressing aberrant GluN3A expression rescues NMDA receptor dysfunction, synapse loss and motor and cognitive decline in Huntington’s disease models. Nat Med.

[12] Spires TL, Grote HE, Garry S, Cordery PM, Van Dellen A, Blakemore C (2004). Dendritic spine pathology and deficits in experience-dependent dendritic plasticity in R6/1 Huntington’s disease transgenic mice. Eur J Neurosci.

[13] Wu J, Ryskamp DA, Liang X, Egorova P, Zakharova O, Hung G (2016). Enhanced store-operated calcium entry leads to striatal synaptic loss in a Huntington’s disease mouse model. J Neurosci.

[14] Cepeda C, Hurst RS, Calvert CR, Hernández-Echeagaray E, Nguyen OK, Jocoy E (2003). Transient and progressive electrophysiological alterations in the corticostriatal pathway in a mouse model of Huntington’s disease. J Neurosci.

[15] Joshi PR, Wu N-P, André VM, Cummings DM, Cepeda C, Joyce JA (2009). Age-dependent alterations of corticostriatal activity in the YAC128 mouse model of Huntington disease. J Neurosci.

[16] Milnerwood AJ, Raymond LA (2007). Corticostriatal synaptic function in mouse models of Huntington’s disease: early effects of huntingtin repeat length and protein load. J Physiol.

[17] Milnerwood AJ, Gladding CM, Pouladi MA, Kaufman AM, Hines RM, Boyd JD (2010). Early increase in extrasynaptic NMDA receptor signaling and expression contributes to phenotype onset in Huntington’s disease mice. Neuron.

[18] Buren C, Tu G, Parsons MP, Sepers MD, Raymond LA (2016). Influence of cortical synaptic input on striatal neuronal dendritic arborization and sensitivity to excitotoxicity in corticostriatal coculture. J Neurophysiol.

[19] Kaufman AM, Milnerwood AJ, Sepers MD, Coquinco A, She K, Wang L (2012). Opposing roles of synaptic and extrasynaptic NMDA receptor signaling in cocultured striatal and cortical neurons. J Neurosci.

[20] Segal M, Greenberger V, Korkotian E (2003). Formation of dendritic spines in cultured striatal neurons depends on excitatory afferent activity. Eur J Neurosci.

[21] Pouladi MA, Stanek LM, Xie Y, Franciosi S, Southwell AL, Deng Y (2012). Marked differences in neurochemistry and aggregates despite similar behavioural and neuropathological features of Huntington disease in the full-length BACHD and YAC128 mice. Hum Mol Genet.

[22] Slow EJ, van Raamsdonk J, Rogers D, Coleman SH, Graham RK, Deng Y (2003). Selective striatal neuronal loss in a YAC128 mouse model of Huntington disease. Hum Mol Genet.

[23] Buren C, Parsons MP, Smith-Dijak A, Raymond LA (2016). Impaired development of cortico-striatal synaptic connectivity in a cell culture model of Huntington’s disease. Neurobiol Dis.

[24] Milnerwood AJ, Kaufman AM, Sepers MD, Gladding CM, Zhang L, Wang L (2012). Mitigation of augmented extrasynaptic NMDAR signaling and apoptosis in cortico-striatal co-cultures from Huntington’s disease mice. Neurobiol Dis.

[25] Cummings DM, Cepeda C, Levine MS (2010). Alterations in striatal synaptic transmission are consistent across genetic mouse models of Huntington’s disease. ASN Neuro.

[26] Ryskamp D, Wu J, Geva M, Kusko R, Grossman I, Hayden M (2017). The sigma-1 receptor mediates the beneficial effects of pridopidine in a mouse model of Huntington disease. Neurobiol Dis.

[27] Van Raamsdonk JM, Pearson J, Rogers DA, Lu G, Barakauskas VE, Barr AM (2005). Ethyl-EPA treatment improves motor dysfunction, but not neurodegeneration in the YAC128 mouse model of Huntington disease. Exp Neurol.

[28] Van Raamsdonk JM, Pearson J, Bailey CDC, Rogers DA, Johnson GVW, Hayden MR (2005). Cystamine treatment is neuroprotective in the YAC128 mouse model of Huntington disease. J Neurochem.

[29] Van Raamsdonk JM, Pearson J, Rogers DA, Bissada N, Vogl AW, Hayden MR (2005). Loss of wild-type huntingtin influences motor dysfunction and survival in the YAC128 mouse model of Huntington disease. Hum Mol Genet.

[30] van Dellen A, Welch J, Dixon RM, Cordery P, York D, Styles P (2000). N-Acetylaspartate and DARPP-32 levels decrease in the corpus striatum of Huntington’s disease mice. Neuroreport.

[31] Bibb JA, Yan Z, Svenningsson P, Snyder GL, Pieribone VA, Horiuchi A (2000). Severe deficiencies in dopamine signaling in presymptomatic Huntington’s disease mice. Proc Natl Acad Sci U S A.

[32] Kolodziejczyk K, Raymond LA (2016). Differential changes in thalamic and cortical excitatory synapses onto striatal spiny projection neurons in a Huntington disease mouse model. Neurobiol Dis.

[33] Kassubek J, Juengling FD, Ecker D, Landwehrmeyer GB (2005). Thalamic atrophy in Huntington’s disease co-varies with cognitive performance: a morphometric MRI analysis. Cereb Cortex N Y N 1991.

[34] Cheng C, Trzcinski O, Doering LC (2014). Fluorescent labeling of dendritic spines in cell cultures with the carbocyanine dye “DiI”. Front Neuroanat.

[35] Miller BR, Bezprozvanny I (2010). Corticostriatal circuit dysfunction in Huntington’s disease: intersection of glutamate, dopamine and calcium. Future Neurol.

[36] Wang N, Gray M, Lu X-H, Cantle JP, Holley SM, Greiner E (2014). Neuronal targets of mutant huntingtin genetic reduction to ameliorate Huntington’s disease pathogenesis in mice. Nat Med.

[37] Estrada-Sánchez AM, Burroughs CL, Cavaliere S, Barton SJ, Chen S, Yang XW (2015). Cortical efferents lacking mutant huntingtin improve striatal neuronal activity and behavior in a conditional mouse model of Huntington’s disease. J Neurosci.

[38] Graveland GA, Williams RS, DiFiglia M (1985). Evidence for degenerative and regenerative changes in neostriatal spiny neurons in Huntington’s disease. Science.

[39] André VM, Cepeda C, Fisher YE, Huynh M, Bardakjian N, Singh S (2011). Differential electrophysiological changes in striatal output neurons in Huntington’s disease. J Neurosci.

[40] Mangiarini L, Sathasivam K, Seller M, Cozens B, Harper A, Hetherington C (1996). Exon 1 of the HD gene with an expanded CAG repeat is sufficient to cause a progressive neurological phenotype in transgenic mice. Cell.

[41] Nithianantharajah J, Barkus C, Vijiaratnam N, Clement O, Hannan AJ (2009). Modeling brain reserve: experience-dependent neuronal plasticity in healthy and Huntington’s disease transgenic mice. Am J Geriatr Psychiatry.

[42] Murmu RP, Li W, Holtmaat A, Li J-Y (2013). Dendritic spine instability leads to progressive neocortical spine loss in a mouse model of Huntington’s disease. J Neurosci.

[43] Lerner RP, Trejo Martinez LDCG, Zhu C, Chesselet M-F, Hickey MA (2012). Striatal atrophy and dendritic alterations in a knock-in mouse model of Huntington’s disease. Brain Res Bull.

[44] Rocher AB, Gubellini P, Merienne N, Boussicault L, Petit F, Gipchtein P (2016). Synaptic scaling up in medium spiny neurons of aged BACHD mice: a slow-progression model of Huntington’s disease. Neurobiol Dis.

[45] Sauvageot CM, Stiles CD (2002). Molecular mechanisms controlling cortical gliogenesis. Curr Opin Neurobiol.

[46] Gomperts SN, Rao A, Craig AM, Malenka RC, Nicoll RA (1998). Postsynaptically silent synapses in single neuron cultures. Neuron.

[47] Boyer C, Schikorski T, Stevens CF (1998). Comparison of hippocampal dendritic spines in culture and in brain. J Neurosci.

[48] Ferrante RJ (2009). Mouse models of Huntington’s disease and methodological considerations for therapeutic trials. Biochim Biophys Acta.

[49] Bayram-Weston Z, Olsen E, Harrison DJ, Dunnett SB, Brooks SP (2016). Optimising Golgi–Cox staining for use with perfusion-fixed brain tissue validated in the zQ175 mouse model of Huntington’s disease. J Neurosci Methods.

