# Supplementary figures and images for: Altering cortical input unmasks synaptic phenotypes in the YAC128 cortico-striatal co-culture model of Huntington disease
Source: BMC Biol. 2018 Jun 27;16:58. doi: 10.1186/s12915-018-0526-3 (PMC6020351; doi:10.1186/s12915-018-0526-3)

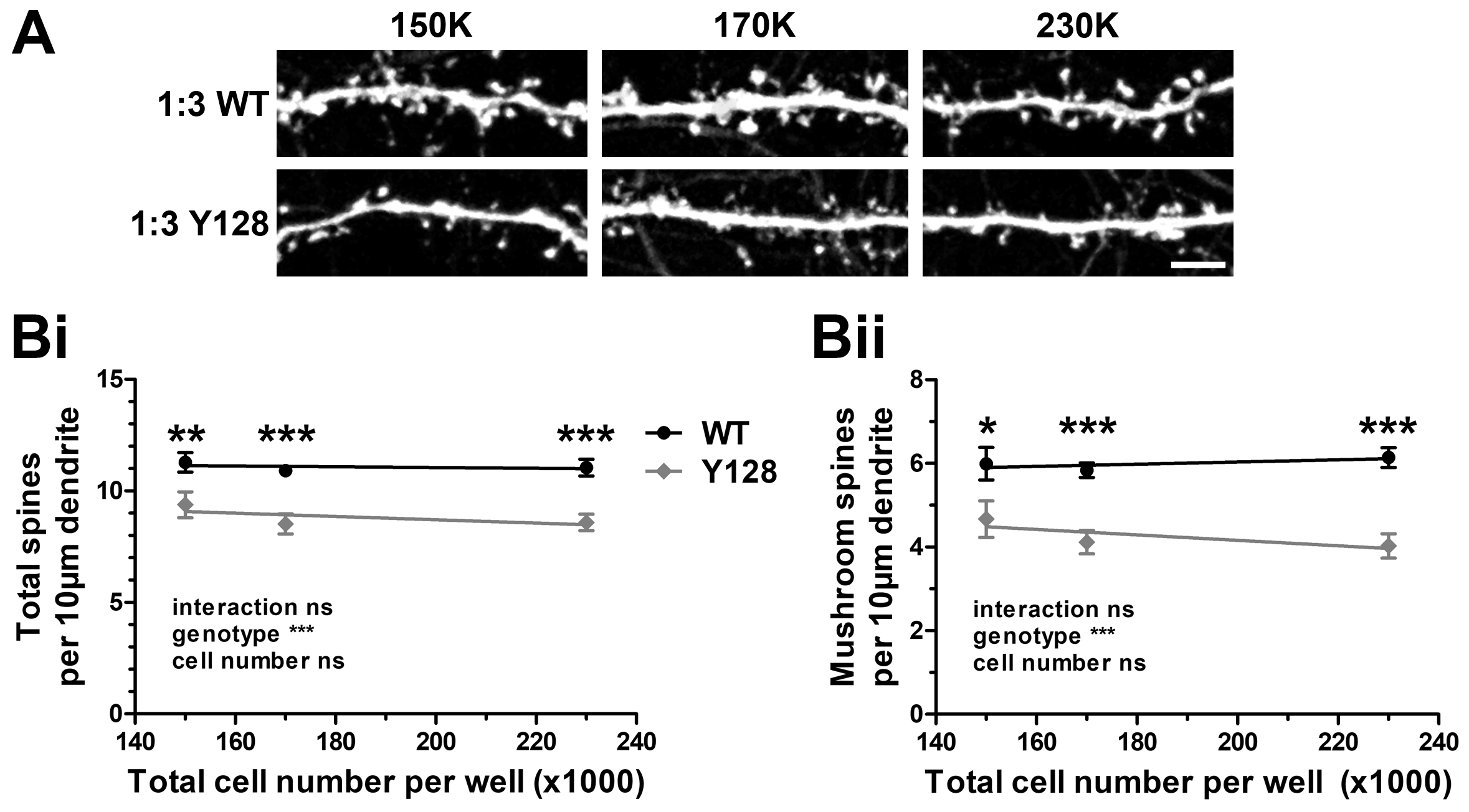

Supplement: Supplementary file 2 — Figure S1. Initial plating density does not impact the presence or severity of YAC128 MSN spine instability. WT and YAC128 co-cultures were generated at a 1:3 CS ratio and plated at three different total cell numbers per well (150,000, 170,000, or 230,000 in 24-well plates). Coverslips were fixed at DIV21 and processed for DARPP32 immunocytochemistry, imaging, and spine analysis. (A) Sample images of DARPP32-stained spines on secondary or tertiary MSN dendrites (scale bar = 5 μm). There was no effect of initial plating density on YAC128 (Bi) total or (Bii) mature mushroom spine density phenotypes [n = 20(2); two-way ANOVA with Bonferroni post-hoc analysis; *p < 0.05, **p < 0.01, ***p < 0.001]. (TIF 423 kb) [file 12915_2018_526_MOESM2_ESM.tif]

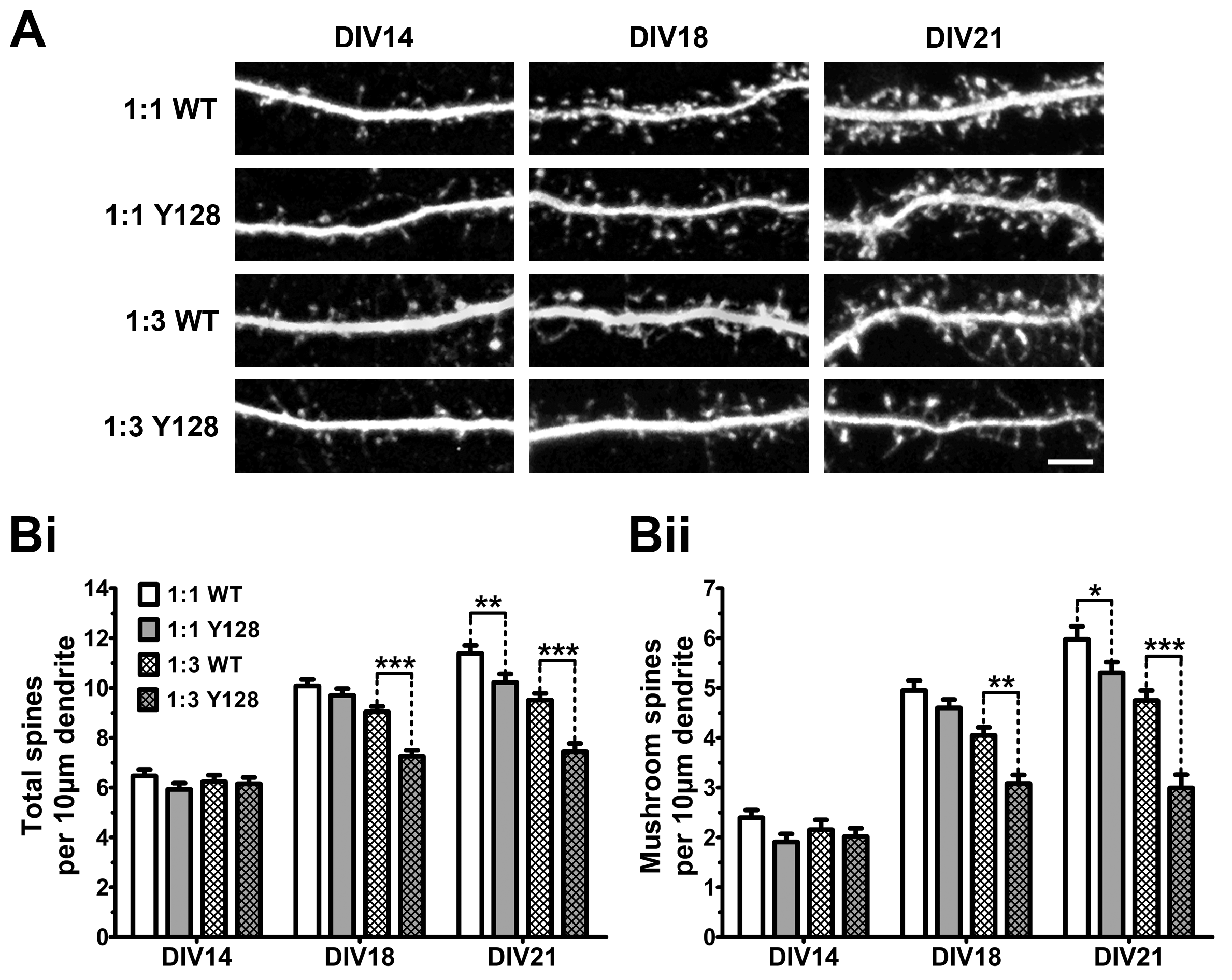

Supplement: Supplementary file 3 — Figure S2. Reduced spine density in co-cultured YAC128 MSNs is a developmental phenotype. WT and YAC128 co-cultures were generated at either a 1:1 or 1:3 CS ratio and processed at DIV14, 18, or 21 for DARPP32 immunocytochemistry, imaging, and spine analysis. (A) Sample images of DARPP32-stained spines on secondary or tertiary MSN dendrites (scale bar = 5 μm). A developmental increase in (Bi) total and (Bii) mature mushroom spine numbers is impaired after DIV14 in co-cultured YAC128 MSNs compared to WT [n = 32(4); two-way ANOVA with Bonferroni post-hoc analysis; *p < 0.05, **p < 0.01, ***p < 0.001]. (TIF 988 kb) [file 12915_2018_526_MOESM3_ESM.tif]

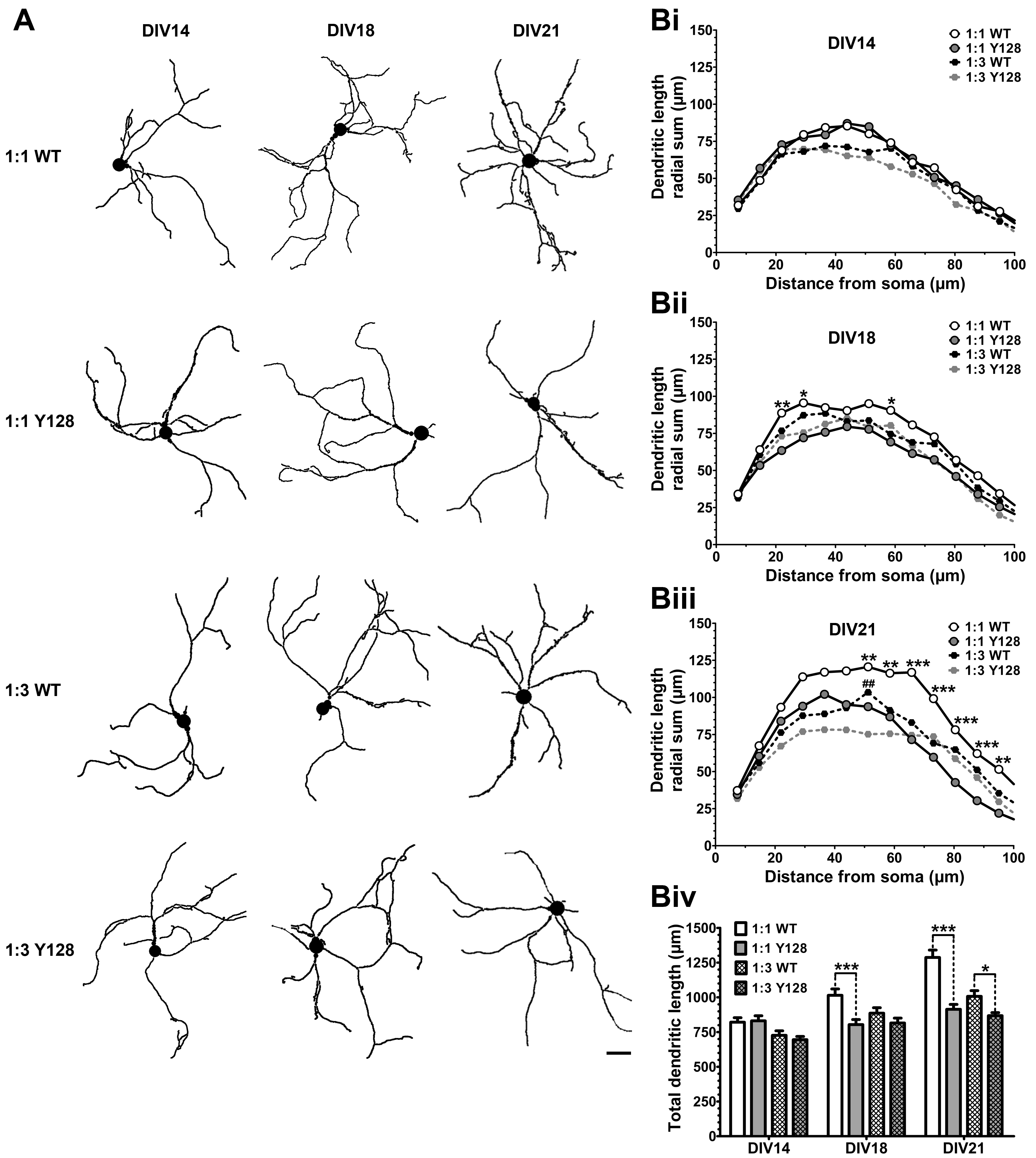

Supplement: Supplementary file 4 — Figure S3. Reduced dendritic length and complexity in co-cultured YAC128 MSNs are developmental phenotypes. WT and YAC128 co-cultures were generated at either a 1:1 or 1:3 CS ratio and processed at DIV14, 18, and 21 for DARPP32 immunocytochemistry, imaging, and dendritic analysis. (A) Sample images of MSN dendritic traces generated in NeuronStudio (scale bar = 15 μm). A developmental increase in (Bi, Bii, Biii) dendritic complexity by Sholl analysis and (Biv) total dendritic length are impaired after DIV14 in co-cultured YAC128 MSNs compared to WT. Post-hoc statistical significance for Sholl analysis is shown only for WT 1:1 vs. YAC128 1:1 (*) or WT 1:3 vs. YAC128 1:3 (#) comparisons [n = 32(4); two-way ANOVA with Bonferroni post-hoc analysis; *p < 0.05, **p < 0.01, ***p < 0.001]. (TIF 875 kb) [file 12915_2018_526_MOESM4_ESM.tif]

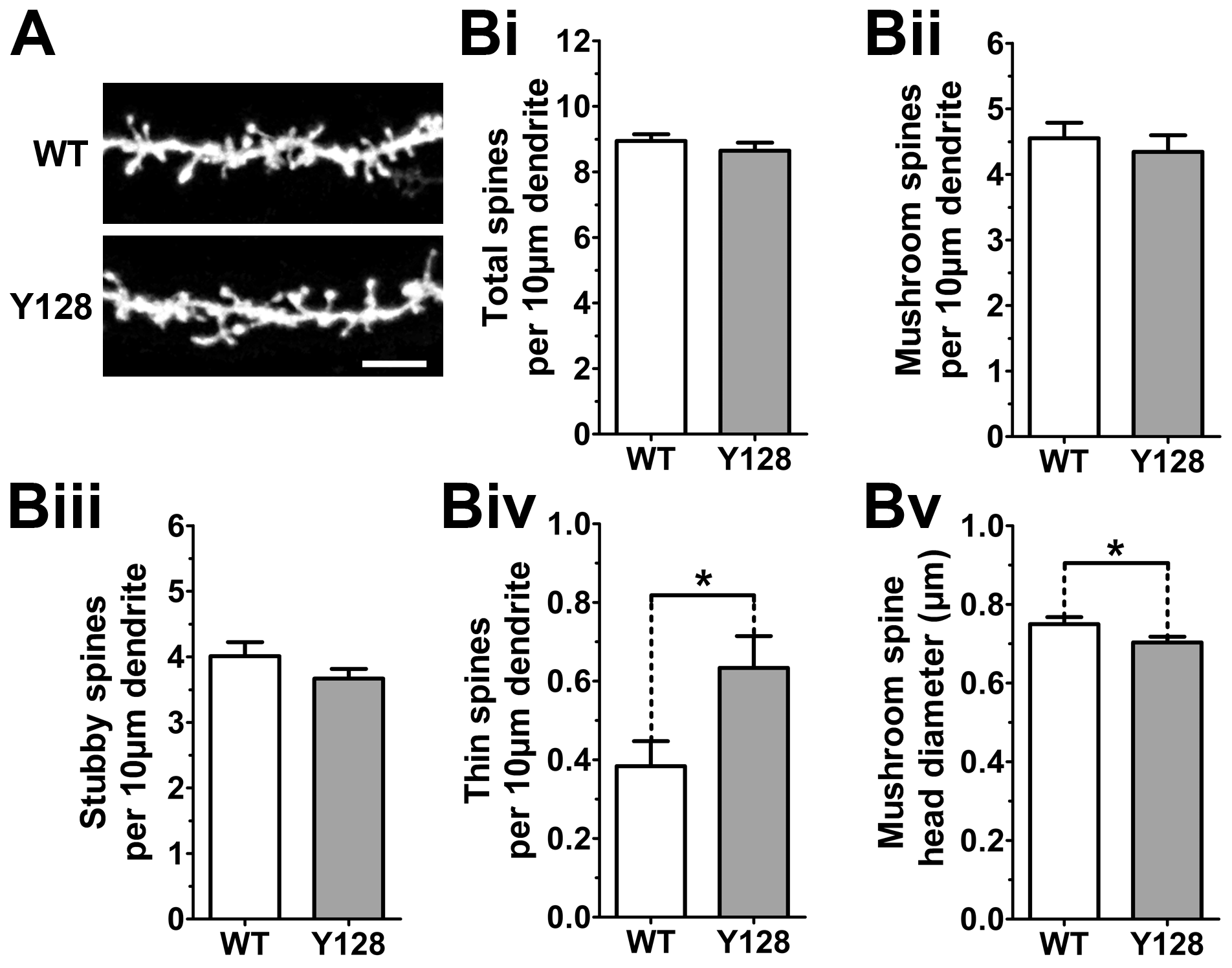

Supplement: Supplementary file 5 — Figure S4. Increased thin spine density and reduced mushroom spine head diameter in DIV21 YAC128 cortical neurons. WT and YAC128 pure cortical cultures were fixed at DIV21 and subjected to in vitro DiI DiOlistic dye labeling for spine analysis. (A) Sample images of DiI-stained spines on cortical dendrites (scale bar = 5 μm). No significant differences in (Bi) total, (Bii) mushroom, or (Biii) stubby spine densities were observed in YAC128 cortical neurons. (Biv) Increased thin spine density and (Bv) reduced mushroom spine head diameter were measured in YAC128 cortical neurons compared to WT [n = 30(3); Student’s t test; *p < 0.05]. (TIF 288 kb) [file 12915_2018_526_MOESM5_ESM.tif]
